# Direct Radiation Model of Louver Shading in Office Building Shade Based on Network Optimization Method

**DOI:** 10.1155/2022/5766448

**Published:** 2022-08-05

**Authors:** Dasi He, Chang Jianguo, Han Xing

**Affiliations:** ^1^School of Energy and Environment, Zhongyuan University of Technology, Zhengzhou 450007, China; ^2^Henan Academy of Building Sciences Co., Ltd, Zhengzhou 450053, China; ^3^PCI Technology Group, Guangzhou 510653, China

## Abstract

In view of the current situation of excessively rapid growth of building energy consumption, a control strategy of external shading louvers based on comprehensive energy consumption is proposed to deeply tap the potential of building energy saving. First, the architectural design software Ecotect is used to establish a simulation model of a shading building in Zhengzhou and import it into the lighting analysis software Daysim and the energy consumption simulation software Energyplus to simulate the annual lighting energy consumption and air-conditioning at 11 kinds of blind angles (15∼165°). For heating energy consumption, take the blind angle corresponding to the minimum comprehensive energy consumption as the opening angle of the dynamic blinds and analyze the comprehensive energy consumption for the whole year under dynamic sunshading. The results show that, compared with the conventional control strategy, the optimized control strategy based on comprehensive energy consumption can greatly improve the building's energy-saving rate.

## 1. Introduction

At present, building energy consumption accounts for about 40.6% of the total energy consumption. With the aggravation of environmental degradation and energy shortage, building energy conservation has attracted more and more attention [1]. The application of shading facilities can control the heat entering the room and reduce the energy consumption of air-conditioning; However, it will inevitably affect the overall indoor illumination and increase lighting energy consumption [2]. Studies have shown that most people spend more than 90% of their time in buildings or cars, so building energy conservation is the top priority in the field of energy conservation. On the premise of meeting the human body's requirements for indoor illumination, improving building energy conservation has become the focus of building design and operation.

In recent years, many scholars have studied the subject of external louver shading. Based on the original sunshade model, Huo et al. established the sunshade correction model of external louver considering the installation spacing and analyzed the influence of installation spacing on the backscattering transmittance of the sunshade model under different louver shading parameters. The results show that under different louver inclination and effective incidence angle, the difference between the two models is obvious, and the proportion of the total transmittance and the amount of backlight of the two models increases with the increase of the reflectivity of the louver surface [3]. Zhang and Wang found that China's energy-saving field mostly focused on the application of energy-saving materials and the improvement of equipment efficiency, but did not consider energy-saving optimization from the design direction [4]. Bunning and Crawford found that different shading angles have a significant impact on energy consumption by studying the impact of shading angles on energy consumption, but the consideration is relatively simple [5]. Liu et al. studied the impact of automatic louver and automatic dimming lighting system on lighting energy consumption and found that the energy-saving rate of north and east lighting can reach 65%, with great energy-saving potential, but did not conduct a comprehensive analysis in combination with the energy consumption of air-conditioning and heating [6].

Taking the office buildings in Zhengzhou as the research object, this paper simulates and calculates the annual lighting energy consumption and air-conditioning and heating energy consumption under 11 kinds of louver inclination angles (15∼165°). The comprehensive energy consumption of rooms in different directions is compared and analyzed, and the control strategy of external sunshade louvers to maximize the energy-saving potential of buildings is explored.

## 2. Theoretical Basis

The thermal simulation method of external sunshade louvers is mainly divided into three stages: input of simulation calculation conditions, solution based on CFD, and analysis of thermal simulation results of sunshade louver.

**Figure 1:**
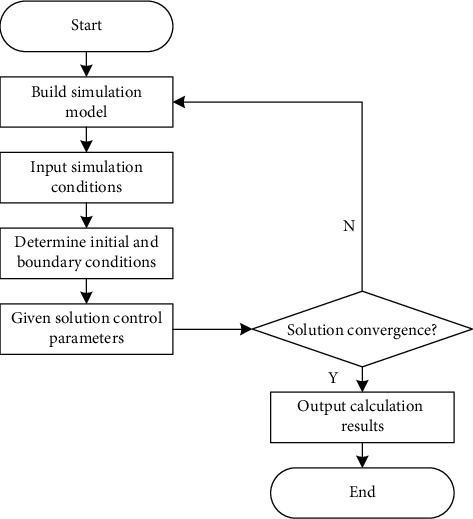
CFD calculation program.

Among them, the input stage of simulation conditions first needs to select the simulation environment, build a 3D model, set simulation parameters, and determine the simulated sunshade louver thermal environment. CFD calculation method has the advantages of simple and easy operation and high accuracy. It can calculate the corresponding value quickly and accurately. Therefore, the energy consumption value after the control of external sunshade louver is calculated according to CFD. The CFD calculation program is shown in Figure 1.

According to the calculation program in Figure 1, the thermal simulation results of external sunshade louvers are obtained, and the building model is established on this basis.

## 3. Project Overview

The test site of this paper is a three storey office building in Zhengzhou. The north-south room size of the office building is 6 × 4 × 3.3 m and window size is 4 × 1.92 m. The size of the east-west room is 4 × 6 × 3.3 m and window size is 2.75 × 1.92 m. The window sill is 0.8 m high and the window wall ratio is 0.4. Louvers are set outside the windows in all directions for shading.

## 4. Technical Design

### 4.1. Building Model

**Figure 2:**
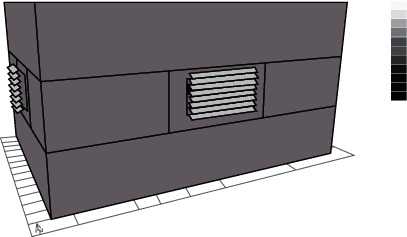
Schematic diagram of building model.

The building model is a three storey office building located in Zhengzhou. As shown in Figure 2, the middle rooms in each direction are taken as the research object to eliminate the influence of the ground, wall, and roof. The parameter settings meet the requirements of GB 50189–2015 design standard for energy efficiency of public buildings [7] and GB 50033–2013 design standard for daylighting of buildings [8].

### 4.2. Description of Simulated Working Conditions

In this paper, the meteorological parameters of Zhengzhou typical meteorological year (CSWD) are selected for simulation calculation, with louver inclination and lighting control type as variables and other parameters as constants.

The ideal air-conditioning system is adopted for the model. The opening time of the air-conditioner is 8 : 00–18 : 00 on working days, the infiltration air volume is 3 times/h, the indoor design temperature in summer is 26°C, the indoor design temperature in winter is 18°C, the personnel density is 0.1 person/m^2^, the lighting power density is 9 W/m^2^, and the electrical power density is 15 W/m^2^. Set the daylighting reference plane 0.75 m from the ground, and the design illuminance is 300 LX according to the requirements of GB 50034–2013 standard for lighting design of buildings [9]. When the natural daylighting illuminance is insufficient, the lighting system is turned on for the supplement.

**Figure 3:**
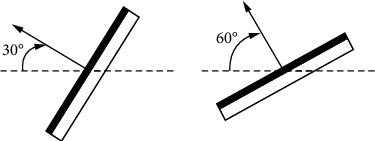
Schematic diagram of louver inclination.

Figure 3 shows the schematic diagram of louver inclination angle with horizontal louver shading side view or vertical louver shading top view.

According to Figure 3, the inclination angle of the louver is defined as the angle between the external normal of the window and the external normal of the louver. Horizontal shading measures shall be taken for rooms facing west and south and vertical shading measures shall be taken for rooms facing east and north. In this way, the control of external sunshade shutters based on network optimization is realized.

## 5. Experimental Analysis

Considering that the louver inclination angles is generally between 0° and 180°, in order to better verify the effectiveness of the proposed control strategy of external sunshade shutter based on comprehensive energy consumption, 11 kinds of louver inclination angles (15∼165°) in the middle are selected as the test objects at an interval of 15°, and the simulation results are output and analyzed.

### 5.1. Energy Consumption Analysis of Air Conditioning and Heating

**Figure 4:**
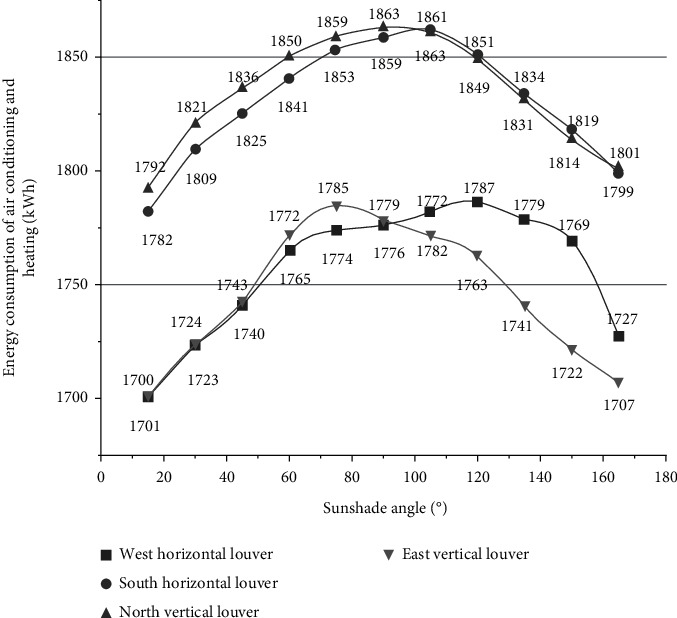
Distribution of energy consumption for air-conditioning and heating in various directions throughout the year.

The building model is imported into Energy Plus to simulate the annual hourly air-conditioning and heating energy consumption.  Figure 4 shows the distribution law of annual air-conditioning and heating energy consumption of rooms facing each direction under 11 kinds of louver inclination angles (15∼165°).

From Figure 4, it is easy to know the change law of air-conditioning and heating energy consumption and the best fixed inclination angle of each room under each shading angle. The shading control mode is changed to dynamic shading based on blocking solar radiation, and then the simulation calculation is carried out. The air-conditioning and heating energy consumption of each room under dynamic shading is 1649 kWh in the west, 1737 kWh in the south, 1753 kWh in the north, and 1654 kWh in the east.

It is easy to know from Table 1 that compared with the best fixed louver shading, dynamic shading has excellent performance in energy saving. The energy-saving rate of air-conditioning and heating in each room has increased by 37.8% in the west, 34.6% in the south, 30.2% in the north, and 35.3% in the east.

### 5.2. Analysis of Lighting Energy Consumption

**Figure 5:**
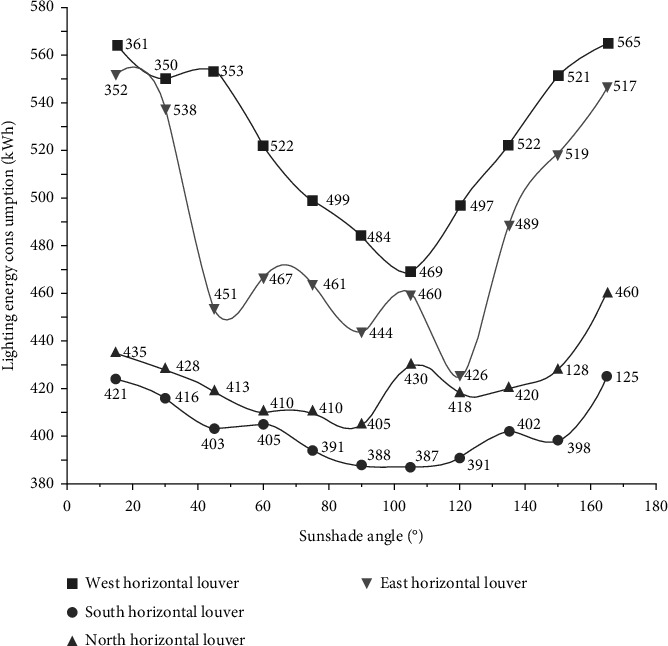
Distribution of annual lighting energy consumption in various directions.

Figure 5 is the annual illumination energy consumption in rooms with different orientations distribution diagram with Daysim output under 11 louver angles (15∼165°).

From Figure 5, it is easy to know the change law of lighting energy consumption towards the room under each shading angle. On the whole, with the increase of shading angle in four directions, it shows a downward trend first and then upward trend. When the west direction is 100°, the minimum energy consumption is 469 kwh, when the south direction is 105°, the minimum energy consumption is 387 kwh, when the north direction is 90°, the minimum energy consumption is 405 kwh, and at 120° to the east, the minimum energy consumption is 426 kwh.

Change the sunshade control mode to dynamic sunshade based on blocking solar radiation, and then conduct simulation calculation to obtain the lighting energy consumption of each facing room combined with two different lighting control systems under dynamic sunshade. The results are shown in Table 1.

It is easy to know from Table 1 that taking the manual switch control alone as the reference, adopting the photoelectric sensor dimming control can further tap the lighting energy-saving potential and increase the lighting energy-saving rate of each room by 16.2% in the west, 18.7% in the south, 19.3% in the north, and 16.8% in the east. This technology is easy to realize by controlling the electric lamp through the dimming mapping level [10].

### 5.3. Comprehensive Energy Consumption Analysis

Without sunshade measures, the comprehensive energy consumption of each room is 2317.5 kWh in the west, 2391.6 kWh in the south, 2407.0 kWh in the north, and 2321.1 kWh in the east. Taking no sunshade as a reference, when adopting the best fixed louver sunshade corresponding to the lowest comprehensive energy consumption, the energy-saving rate of buildings in each direction is 5.8% in the west, 5.4% in the south, 5.4% in the north, and 5.7% in the east. Taking the louver inclination corresponding to the minimum hourly comprehensive energy consumption of the whole year as the dynamic louver opening angle for simulation calculation, the annual comprehensive energy consumption is obtained.

**Figure 6:**
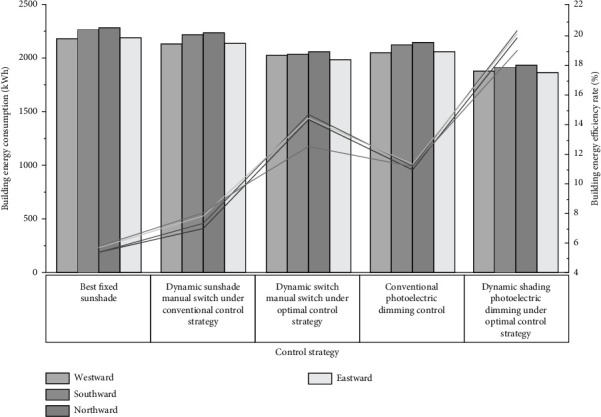
Energy saving effect diagram of each orientation under different control strategies.

In order to make it easy to distinguish, the dynamic shading control strategy based on blocking solar radiation is called conventional control strategy, and the control strategy of taking the louver inclination corresponding to the minimum hourly comprehensive energy consumption throughout the year as the dynamic louver opening angle is called optimal control strategy.  Figure 6 shows the effect of building energy conservation under different control strategies for each facing room.

It is easy to know from Figure 6 that compared with the conventional control strategy, the optimized control strategy can greatly improve the building energy-saving rate. Among them, when the lighting system is controlled by manual switch alone, the energy-saving rate of buildings facing rooms can be increased by 56.2% in the west, 101.5% in the south, 105.4% in the north, and 85.6% in the east. When the lighting system adopts manual switch + photoelectric sensor dimming control, the energy-saving rate of buildings facing rooms can be increased by 65.9% in the west, 82.8% in the south, 81.4% in the north, and 77.3% in the east.

It can also be seen from Figure 6 that when the lighting system adopts manual switch + photoelectric sensor dimming control, the building energy-saving potential can be further tapped. Under the conventional control strategy, compared with the manual switch control of the lighting system alone, the energy-saving rate of rooms in each direction can be further improved by 42.1% in the west, 51.1% in the south, 55.8% in the north, and 45.4% in the east. Under the optimized control strategy, compared with the lighting system adopting manual switch control alone, the energy-saving rate of rooms in each direction can be further improved by 51.0% in the west, 37.2% in the south, 37.7% in the north, and 38.9% in the east.

**Figure 7:**
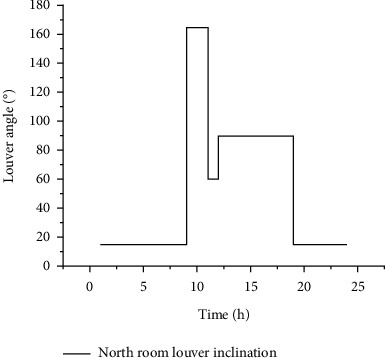
Dynamic louver inclination schedule.

In this paper, the dynamic louver inclination schedule of each direction is obtained by using the above simulation results. The method is as follows: during working hours, in the air-conditioning and heating season, the louver inclination corresponding to the minimum comprehensive energy consumption is taken as the dynamic louver opening angle. In the transition season, the louver angle corresponding to the minimum lighting energy consumption is taken as the dynamic louver opening angle. During nonworking hours, air-conditioning, heating, and transition seasons should be conducive to heat dissipation, heat preservation, and ventilation respectively. Therefore, the louver inclination when the louver is vertical, parallel, and perpendicular to the outer window is taken as the dynamic louver opening angle.  Figure 7 shows the dynamic louver inclination schedule of north facing room in winter solstice under the control of dynamic shading and photoelectric dimming lighting system under the optimal control strategy.

Based on the above simulation results, when the lighting system is controlled by manual switch alone, the energy-saving rate of rooms in each direction can be increased by 56.2% in the west, 101.5% in the south, 105.4% in the north, and 85.6% in the east. When the lighting system adopts manual switch + photoelectric sensor dimming control, the energy-saving rate of buildings facing rooms can be increased by 65.9% in the west, 82.8% in the south, 81.4% in the north, and 77.3% in the east. After adopting the optimized control strategy in this paper, compared with the lighting system adopting manual switch control alone, the energy-saving rate of rooms in each direction can be further improved by 51.0% in the west, 37.2% in the south, 37.7% in the north, and 38.9% in the east.

## 6. Conclusion

This paper simulates, analyzes, and compares the energy consumption of air-conditioning, heating, and lighting in the typical working period of office space in cold areas of China and explores the control strategy of external sunshade louver to maximize the building energy-saving potential. The simulation analysis shows that the optimal control strategy based on comprehensive energy consumption proposed in this paper can greatly improve the building energy efficiency, and the control strategy has important reference significance for new building design. When the lighting system adopts manual switch + photoelectric sensor dimming control, the building energy-saving potential can be further tapped. This conclusion has certain reference significance for the establishment of energy-saving system.

## Figures and Tables

**Table 1:** Lighting energy consumption.

Control type	Manual switch (kW)	Manual switch + photoelectric dimming (kW)
Westward	482.5	404.2
Southward	479.6	390.1
Northward	486.0	392.2
Eastward	487.1	405.4

## Data Availability

The data used to support this study are available from the corresponding author upon request.
